# Disarming COX-1 to disrupt Alzheimer’s inflammatory trajectory: preclinical insights and translational promise

**DOI:** 10.1186/s40035-025-00509-1

**Published:** 2025-09-02

**Authors:** Gilda Loffredo, Marcello D’Amelio

**Affiliations:** 1 Department of Medicine and Surgery, Università Campus Bio-Medico di Roma, Rome, Italy; 2 Wyss Center for Bio and Neuro Engineering, Geneva, Switzerland; 3 Department of Experimental Neurosciences, IRCCS Santa Lucia Foundation, Rome, Italy

## Main text

Alzheimer’s disease (AD) is now widely recognized as a multi-circuit, multifactorial disorder rather than a pure proteinopathy [1]. While the classical hallmarks amyloid-beta (Aβ) deposition and tau hyperphosphorylation remain diagnostic cornerstones, neuroinflammation, primarily driven by microglia, has emerged as a core disease engine [2]. Under pathological conditions, microglia shift from homeostatic to reactive phenotype, releasing cytokines, chemokines, and prostaglandins that exacerbate neuronal dysfunction. These inflammatory mediators are primarily produced via the enzymatic activity of cyclooxygenases (COXs). Although COX-2 has long been the focus of inflammation-related studies in neurodegeneration, COX-1—a constitutively expressed enzyme traditionally considered as a "housekeeping" one—is increasingly recognized for its involvement in microglial activation and neuroinflammatory processes in neurodegenerative disorders such as AD [3].

A recent study by Wang et al., published in *Translational Neurodegeneration*, provides compelling preclinical evidence implicating COX-1 as a key modulator of microglial-driven inflammation in AD. Notably, COX-1 expression was found significantly upregulated in AD and predominantly localized to microglia. This cell-specific expression pattern was evident not only in the 5xFAD mouse model of familial AD but also in human AD brains, as demonstrated by single-cell transcriptomic data from the ssREAD and ROSMAP cohorts. Notably, COX-1 expression increased in a stage-dependent manner, rising from normal aging to clinical AD, thereby reinforcing its pathological relevance. Astrocytes and neurons, in contrast, exhibited minimal or undetectable expression of COX-1, respectively.

Preclinical results from COX-1 knock-out (KO) 5xFAD mice also indicated that genetic deletion of COX-1 markedly reduced neuroinflammatory signaling, Aβ accumulation, and cognitive decline. These findings not only challenge long-standing assumptions about the role of COX-1 in the brain, but also identifies it as a promising therapeutic target for AD [4].

Functionally, COX-1 KO 5xFAD mice show preserved microglial morphology and significant reductions of microglial proliferation and expression of proinflammatory markers such as NOD-like receptor 3 (NLRP3), interleukin-1β (IL-1β), and CD68. Moreover, these mice displayed a similar decrease in Aβ deposition in both the hippocampus and the cortex. This cellular improvement was paralleled by enhanced performance in spatial learning and memory assessments, underscoring the mechanistic involvement of COX-1 in both neuroinflammation and cognitive decline.

Further investigation identified the prostaglandin E2 (PGE_2_)/EP2 receptor (EP2R) signaling axis as a downstream effector of COX-1 activity. Activation of this pathway increases cyclic adenosine monophosphate (cAMP) levels, triggers protein kinase A (PKA) activation, and promotes nuclear translocation of NF-κB, ultimately leading to NLRP3 inflammasome assembly. In vitro analysis also indicated that inhibition of COX-1 and consequently its downstream signaling, replicated the neuroprotective effects observed in COX-1 KO 5xFAD mice, suggesting a potentially druggable pathway.

Importantly, long-term COX-1 deletion did not result in adverse outcomes such as spontaneous microhemorrhages or systemic toxicity. While bleeding time was modestly extended, blood–brain barrier integrity and organ function remained unaffected, suggesting the safety of sustained COX-1 inhibition in chronic disease contexts.

Notwithstanding these encouraging preclinical data, past attempts to translate COX-1 inhibition into meaningful clinical benefit for patients with AD have shown mixed results. In a double-blind, placebo-controlled trial, indomethacin failed to slow cognitive decline over six months, and > 20% of participants discontinued treatment because of gastrointestinal toxicity [5]. A subsequent one-year study involving 51 mild-to-moderate AD patients similarly showed inconclusive results in slowing the disease progression [6]. Triflusal, a COX-1 inhibitor chemically-related to aspirin, was associated with reduced conversion from amnestic mild cognitive impairment (MCI) to AD [7], but this benefit was not replicated in established AD and was accompanied by bleeding risks. These results highlight the limitations of first-generation COX-1 inhibitors that act peripherally or have poor brain-penetration, underscoring the need for next-generation agents with enhanced selectivity, pharmacokinetics, and cerebral exposure.

However, COX-1 downstream signaling components could be attractive therapeutic targets. Selective EP2R antagonists may offer comparable efficacy with a lower risk of bleeding-related side effects [8]. Indeed, rational development of highly selective, brain-penetrant COX-1 or EP2R inhibitors that minimize peripheral prostaglandin blockade represents a promising “second chance” to revisit this pathway in earlier disease stages, such as MCI or pre-symptomatic carriers, where neuroinflammatory amplification may be most amenable to intervention.

Beyond its role in neuroinflammation, the study suggests a potential intersection between the COX-1-EP2-cAMP axis and dopaminergic pathways. Although dopamine (DA) signaling was not directly investigated, previous studies demonstrated that DA modulates neuroinflammatory responses via its receptors (DRs), which are expressed on both microglia and astrocytes [9]. Interestingly, both COX-1 and DA pathways converge on intracellular messengers like cAMP and PKA, implying a possible intersection in their signaling mechanisms.

**Fig. 1:**
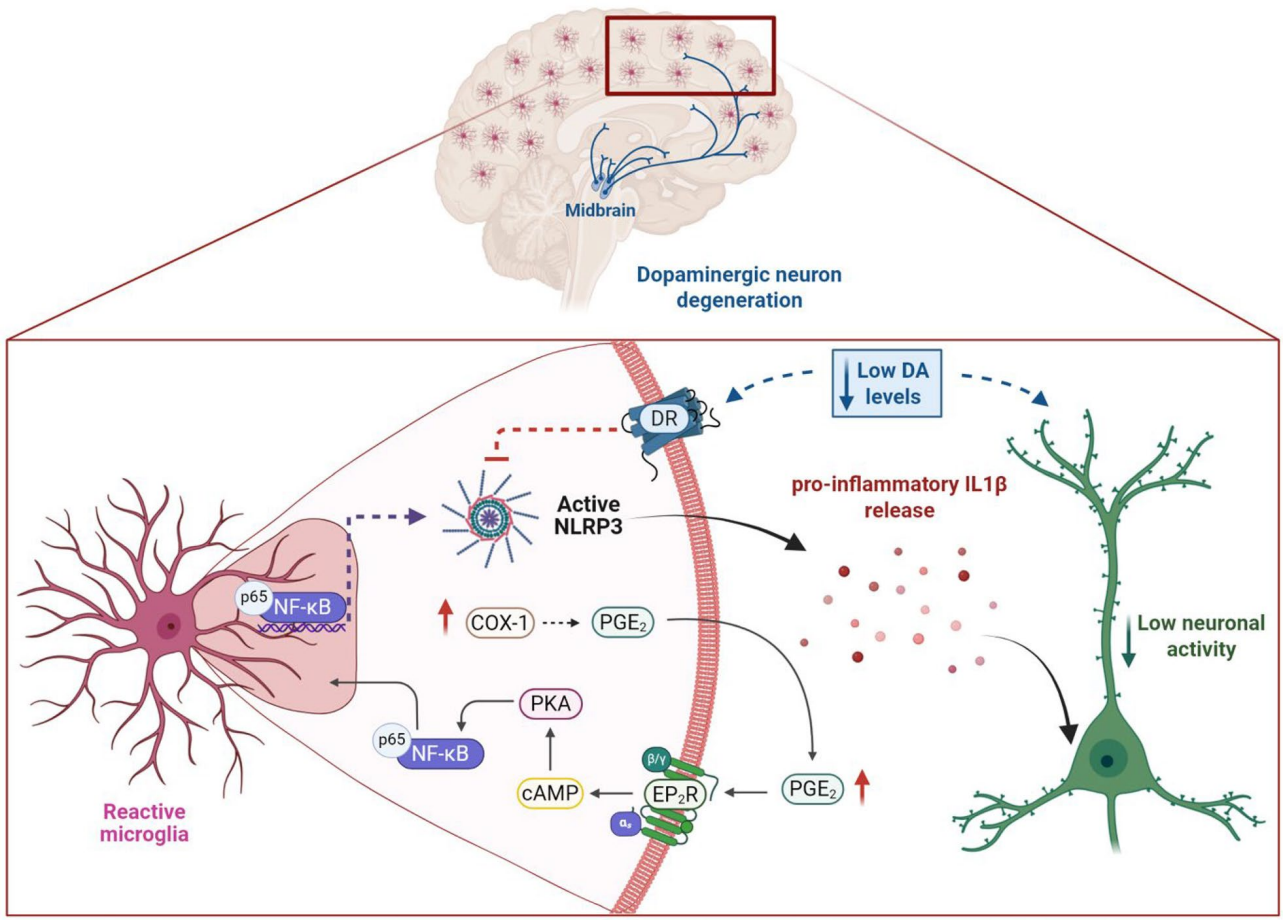
Combined COX-1 upregulation and reduced dopaminergic tone could drive NLRP3 inflammasome activation in Alzheimer’s disease. Schematic representation of neuroinflammatory signaling in AD brain. Reactive microglia upregulate cyclooxygenase-1 (COX-1), leading to increased prostaglandin E₂ (PGE₂) production and release. Once released, PGE₂ acts in paracrine and autocrine pathways by binding to EP2 receptor (EP2R) on microglia and neighboring cells. The engagement of EP2R activates the cAMP–PKA signaling cascade, culminating in NF-κB nuclear translocation. In the nucleus, NF-κB promotes the transcription of *NLRP3* gene, which drives inflammasome assembly and the subsequent release of pro-inflammatory cytokines (e.g. IL-1β). In parallel, a reduction in DA receptor (DR) signaling—as observed in AD—may exacerbate microglial activation and directly increase neuronal vulnerability. Together, dysregulation of the COX-1/PGE₂–EP2 axis and weakened dopaminergic tone may establish a feed-forward loop that perpetuates chronic neuroinflammation and accelerates neurodegeneration in AD. This figure was created with BioRender (https://biorender.com/)

In AD, where dopaminergic deficits and microglial activation commonly co-occur, COX-1 upregulation is particularly pronounced [10–13]. Preclinical evidence from an AD mouse model demonstrates that early degeneration of ventral tegmental area (VTA) DA neurons occurs at pre-plaque stages, resulting in significant memory impairments and disrupted reward processing [12]. These findings highlight the critical role of dopaminergic dysfunction in the early stages of AD, before the onset of Aβ plaque formation [12]. Consistent with this, functional neuroimaging studies in patients have revealed disruption of DA-related connectivity across the AD spectrum, particularly within the mesocorticolimbic pathway—including key regions such as the ventral striatum and the hippocampus. These connectivity disruptions correlate with early cognitive decline and region-specific brain atrophy [13]. Furthermore, in experimental models of Parkinson’s disease, the majority of PGE_2_ in the midbrain is derived from COX-1, rather than COX-2 [14]. Given that DR activation can blunt NLRP3 activity [9, 15], a reduction in dopaminergic tone may potentiate COX-1-driven inflammation—or both systems may converge on a shared inflammatory cascade (Fig. 1). Elucidating this crosstalk could offer critical insights into overlapping neuroimmune pathways in AD and related disorders.

This study clearly establishes microglial activation as a validated and translationally actionable target in AD. Characterization of the COX-1-PGE_2_-EP2R pathway (Fig. 1) provides a convincing molecular framework for modulating neuroinflammatory pathways that contribute to both Aβ pathology and cognitive impairment. However, integrating the mixed clinical record of COX-1 inhibitors tempers unbridled optimism and emphasizes the importance of refining compound properties and patient-selection strategies. Crucially, the safety profile observed with long-term COX-1 deletion reinforces its potential for chronic therapeutic intervention.

By shifting focus from the traditionally emphasized COX-2 to COX-1, this work also redefines the neuroinflammatory landscape in AD and introduces a previously overlooked target for precision medicine. The successful reduction of neuroinflammation and Aβ accumulation, and the amelioration of cognitive functions through both genetic and pharmacologic strategies, identify COX-1 and its downstream effectors as attractive candidates for disease-modifying therapies. Future advancements in treatment will depend on next-generation, brain-optimized inhibitors and clinical trials that intervene earlier in the disease course, where anti-inflammatory mechanisms may yield the greatest impact.

Moreover, the potential convergence between COX-1 signaling and dopaminergic pathways highlights an emerging intersection of neuroimmune and neuromodulatory dysfunction in AD. Although speculative, this link requires further investigation, as it may provide a basis for integrated therapeutic strategies that simultaneously restore neurotransmitter balance and dampen neuroinflammation.

Future studies should aim at validating these findings in additional rodent AD models and human tissues, exploring the therapeutic efficacy of selective EP2 antagonists in clinical settings, and dissecting the molecular interaction between COX-1 activity and dopaminergic signaling. Ultimately, such multi-targeted approaches may offer novel opportunities to intercept the disease progression, improve cognitive outcomes, and tailor treatment to individual AD patients.

## Data Availability

Not applicable.

## Abbreviations

AD: Alzheimer’s disease
Aβ: Amyloid-β
cAMP: Cyclic adenosine monophosphate
COXs: Cyclooxygenases
DA: Dopamine
DRs: Dopamine receptors
IL-1β: Interleukin-1β
KO: Knock-Out
MCI: Mild cognitive impairment
NLRP3: NOD-like receptor 3
PGE_2_: Prostaglandin E2
EP2R: EP2 receptor
PKA: Protein kinase A
